# Unraveling the Potential of Black Soldier Fly Larvae as a Sustainable Protein Source for Nile Tilapia Production in Diverse Aquaculture Systems

**DOI:** 10.1155/anu/3598843

**Published:** 2025-01-17

**Authors:** Jonathan Munguti, Mavindu Muthoka, Jimmy B. Mboya, Domitila Kyule, Menaga Meenakshisundaram, Chrysantus M. Tanga

**Affiliations:** ^1^Kenya Marine and Fisheries Research Institute (KMFRI), National Aquaculture Research Development and Training Centre (NARDTC), Sagana, Kenya; ^2^Department of Animal and Fisheries Sciences, Maseno University, Maseno, Kenya; ^3^International Centre of Insect Physiology and Ecology, Nairobi, Kenya

**Keywords:** aquaculture, black soldier fly larvae meal, *Hermetia illucens*, Nile tilapia, nutritional composition, sustainable aquafeeds

## Abstract

Aquaculture plays a critical role in global food security, with Nile tilapia (*Oreochromis niloticus*) recognized for its adaptability and robust growth. However, traditional feeds, heavily reliant on fishmeal (FM) and soybean meal, face economic and environmental challenges. In response, black soldier fly larvae meal (BSFLM) has emerged as a promising, nutrient-dense alternative. This review synthesizes existing literature on BSFLM's nutritional profile and its suitability for Nile tilapia diets, while acknowledging that the data come from diverse independent studies conducted under varying environmental conditions and husbandry practices. BSFLM consistently provides high-quality protein (29.9%–48.2%), aligning with tilapia's requirements, and is rich in essential minerals and fatty acids. While its lipid content (25.69%–28.43%) may require processing adjustments, the overall profile supports tilapia health and growth. Trends from case studies suggest that certain systems, such as hapas placed in ponds, have reported favorable growth and feed conversion efficiencies at 50% FM replacement levels. However, these findings cannot be directly compared across all studies due to differences in methodologies, culture conditions, and inclusion rates. Instead, they collectively indicate that BSFLM can effectively replace traditional protein sources and enhance sustainability. As research and production scale up, careful consideration of context, system design, and feed formulations will be essential. Collaborative efforts among researchers, industry, and policymakers will further refine the use of BSFLM, ultimately advancing the environmental and economic sustainability of Nile tilapia aquaculture.

## 1. Introduction

Aquaculture has become an increasingly vital sector in global food production, addressing the rising demand for animal protein [1]. It plays a crucial role in food security, particularly in developing countries where it provides a significant source of livelihood and nutrition. As the fastest-growing food production sector, aquaculture contributes to nearly half of the global fish supply, and this share is expected to rise in the coming decades [2]. Among the various species cultivated in aquaculture, Nile tilapia (*Oreochromis niloticus*) is one of the most important [3]. The species is a cornerstone of global aquaculture, widely recognized for its adaptability, rapid growth rates, and resilience to various environmental conditions. This species has become one of the most farmed fish worldwide, playing a critical role in enhancing food security, particularly in developing countries where it also serves as a significant source of income [4]. In 2022, global production of Nile tilapia was ~5 million tons, ranking second after grass carp among farmed finfish [5], underscoring its importance in the aquaculture sector. The species' ability to thrive in a wide range of culture systems—including traditional pond systems, recirculating aquaculture systems (RAS), biofloc systems, and cage cultures—has made it a versatile choice for aquaculturists around the globe [6]. Moreover, Nile tilapia's omnivorous diet and low trophic level have further solidified its status as a sustainable protein source, essential for meeting the growing global demand for animal protein, especially in regions with limited resources [7].

Despite the widespread success of Nile tilapia farming, the industry is confronted with significant challenges, particularly in the realm of feed costs and environmental sustainability [8]. Conventional aquaculture feeds, primarily composed of fishmeal (FM) and soybean meal, are becoming increasingly unsustainable both economically and ecologically [9]. FM, derived from wild-caught fish, is not only expensive but also contributes to the depletion of marine ecosystems, raising concerns about the long-term viability of relying on this resource [10]. The pressure on FM supplies is exacerbated by the growing demand from the expanding aquaculture sector, making it a critical bottleneck in the sustainable expansion of the industry [10]. Soybean meal, while less costly, presents its own set of challenges, including its association with deforestation, loss of biodiversity, and competition with food crops for arable land [11]. These issues have intensified the search for alternative protein sources that can mitigate environmental impacts while maintaining or improving production efficiency.

In response to these challenges, insect-based feeds have emerged as a promising alternative. Insect meals, particularly black soldier fly (*Hermetia illucens*) larvae meal (BSFLM), are particularly attractive due to their high protein content, favorable lipid profiles, and the presence of essential amino acids (EAAs) that are comparable to those found in FM [12]. Moreover, insects are a natural component of the diet for many fish species, including Nile tilapia, making them a biologically appropriate feed source [13]. The environmental benefits of insect farming are substantial: it requires significantly less land and water than traditional protein sources, and insects can be reared on organic waste, converting low-value byproducts into high-quality feed ingredients [14]. This reduces the environmental footprint of feed production and contributes to waste management solutions.

Despite these advantages, its widespread adoption in aquaculture has been limited by high production costs associated with large-scale rearing and processing which make BSFLM less economically competitive compared to traditional feed ingredients [15]. Additionally, public perception and consumer acceptance play significant roles, as some consumers may be hesitant about the use of insects in animal nutrition [16]. Regulatory challenges also pose obstacles, with varying standards and approvals required across different regions, complicating the commercialization of BSFLM-based feeds. Furthermore, the lack of standardized production and processing protocols can result in inconsistencies in feed quality, hindering its integration into mainstream aquaculture practices [17]. The adoption of BSFL-based fish feeds in aquaculture has also been slow, partly due to the need for more research on their efficacy across different aquaculture systems.

While several studies have indeed evaluated the effects of FM replacement with BSFLM on growth-related parameters of Nile tilapia (e.g., weight gain, specific growth rate [SGR], feed conversion ratio [FCR], and survival), these comparisons are often confined to single experimental systems—such as hapas, cages in earthen ponds, glass aquaria, or plastic tanks—while varying other factors like environmental or feeding conditions [16, 18, 19]. Consequently, although we have data on growth performance parameters, there remains a scarcity of information on how these indicators compare across multiple, diverse culture systems. Differences in methodologies, culture conditions, and inclusion rates further limit direct comparisons between studies. In this review, we acknowledge these constraints and focus on highlighting trends rather than absolute comparisons. The aim is to provide a foundation of knowledge that, while derived primarily from controlled experimental environments, can guide further research into more complex, large-scale commercial systems such as RAS, aquaponics, ponds, and cages. Ultimately, understanding the potential of BSFLM in a broader range of production scenarios will inform more sustainable and efficient feeding strategies for Nile tilapia.

## 2. Methodology

The literature search for this review was conducted with the primary aim of collating and synthesizing existing research on the use of insect-based feeds in Nile tilapia aquaculture across different culture systems. A systematic approach was employed to ensure the inclusion of relevant and high-quality studies. The search process involved multiple academic databases, including Web of Science, Scopus, PubMed, and Google Scholar, to capture a wide range of peer-reviewed articles, conference papers, and relevant gray literature. The search strategy was carefully developed to encompass all relevant aspects of the review topic. Keywords were selected to reflect the core elements of the study, including “Nile Tilapia,” “*Oreochromis niloticus*,” “insect-based feeds,” “black soldier fly larvae,” “mealworm,” “aquaculture systems,” “recirculating aquaculture systems,” “pond,” “aquaponic,” and “cage culture.” Boolean operators such as “AND” and “OR” were used to refine the search queries, and truncation techniques were applied to capture various word forms and synonyms.

The inclusion criteria for the literature were clearly defined to ensure the relevance and quality of the studies selected. Studies were included if they met the following criteria: (1) they focused on Nile tilapia as the primary species, (2) they evaluated the use of BSFLM as a replacement or supplement for traditional feeds, (3) they provided data on growth performance, FCRs, health outcomes, or water quality. Additionally, only studies published in English and within the last 20 years (2004–2024) were considered to ensure the review's relevance to current aquaculture practices.

**Figure 1:**
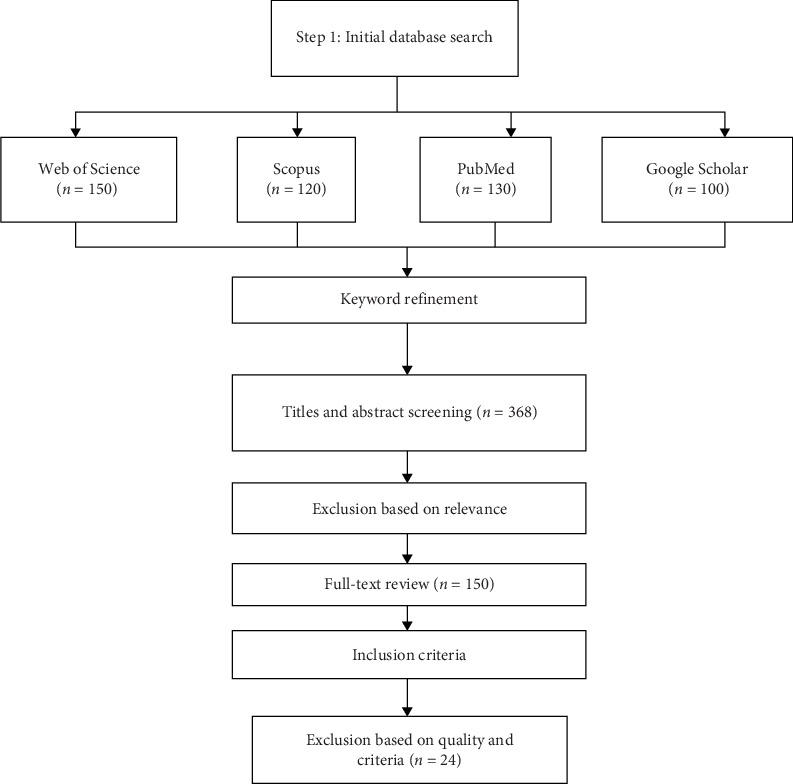
Literature search strategy.

Exclusion criteria were also established to maintain the focus and rigor of the review. Studies were excluded if they (1) did not involve Nile tilapia, (2) focused on BSFL-based fish feeds without a direct comparison to conventional feeds, (3) lacked empirical data or were purely theoretical, or (4) were not accessible through institutional subscriptions or open-access platforms. The initial search yielded over 500 articles, which were then screened for relevance based on titles and abstracts. This screening process was followed by a full-text review of 150 articles, resulting in the selection of 24 studies that met all inclusion criteria for detailed analysis (Figure 1).

## 3. Nutritional Composition and Benefits of BSFLM in Nile Tilapia Diet

### 3.1. Primary Macronutrient Composition

The nutritional profile of BSFLM (Table 1) demonstrates significant potential as a sustainable protein source for Nile tilapia feeds, although its crude protein (CP) content, which ranges between 29.9% and 48.2% [20–22], is lower than that typically found in FM (55.1%–62.6% CP; [25, 26]). While these BSFLM protein levels fall within or near the dietary CP requirements for tilapia at various stages—fry and juveniles (30%–56% CP) and larger fish (28%–30% CP) [23]—fully replacing FM (≥55.1% CP) with BSFLM (≤48.2% CP) may not provide enough protein on its own. Instead, a strategic partial replacement of FM with BSFLM, potentially supplemented by other protein sources or amino acids, can still meet the required CP levels. Moreover, the variability in BSFLM protein content likely arises from differences in larval rearing conditions, diets, and processing methods, and optimizing these factors can further improve its suitability as a protein ingredient for Nile tilapia feeds.

In addition to protein, the lipid content of BSFLM also presents an interesting advantage for formulating tilapia diets. BSFLM has a crude lipid content ranging from 25.69% to 28.43% [20–22], which is notably higher than the typical lipid requirements of Nile tilapia. For fry up to 2.5 g, the optimum dietary lipid concentration is about 5.2%, decreasing to 4.4% as they reach 7.5 g, and further decreasing to 6%–8% for larger fish [23]. Although the lipid content in BSFLM exceeds these levels, this could be managed through feed formulation strategies, such as blending BSFLM with lower-fat ingredients or defatting the larvae before incorporation into the feed. Excessive dietary lipids can lead to fat deposition in fish, which may negatively affect growth performance and carcass quality. Therefore, careful attention to lipid management is essential when utilizing BSFLM in tilapia feeds. Defatting processes could help meet the specific lipid needs of Nile tilapia and improve the overall digestibility and energy balance in the diet.

Another important component of BSFLM is its crude fiber content, which ranges from 9.48% to 9.96%. While fiber plays a role in promoting gut health and facilitating digestion, excessive amounts can reduce nutrient absorption and feed efficiency in fish. Nile tilapia, like many other fish species, are not highly efficient at digesting high levels of fiber, and optimal feed formulations typically limit fiber content [24]. Therefore, the relatively high fiber content in BSFLM may require adjustments during feed formulation to avoid negative impacts on nutrient availability. Strategies such as partial inclusion of BSFLM or processing methods like enzymatic treatment can help reduce the fiber content, making it more suitable for tilapia diets without compromising growth performance [27].

Moisture content, which ranges between 3.21% and 7.10% [20–22], is another consideration for feed stability and shelf life. High moisture levels in feed ingredients can lead to spoilage and reduced shelf stability, which is especially critical in warm and humid aquaculture environments. The relatively low moisture content of BSFLM, particularly after processing, makes it a stable ingredient for long-term storage and use in aquaculture feeds. This enhances its practicality in commercial feed production, where storage and transportation conditions can significantly affect feed quality.

The ash content of BSFLM ranges from 6.7% to 15.4% [20–22]. While tilapia require adequate minerals for skeletal development and overall health, high ash content in feed can dilute the energy density and nutrient content, potentially leading to lower growth efficiency. However, the mineral profile of BSFLM can be beneficial, particularly in calcium and phosphorus supplementation, which are critical for bone development in fish [28].

### 3.2. Vitamin Composition of BSFLM and Their Nutritional Requirements in Nile Tilapia

Vitamins play a crucial role in the health, growth, and metabolic functions of Nile tilapia, as they are involved in processes such as energy production, immune function, and tissue maintenance [29]. BSFLM has been identified as a potential source of essential nutrients, including vitamins, which have been discussed and summarized in Table 2.

#### 3.2.1. Vitamin B1 (Thiamin)

Thiamin (vitamin B1) is an essential coenzyme in the metabolism of carbohydrates, facilitating the conversion of carbohydrates into energy via decarboxylation and transketolase reactions [40]. In fish, thiamin is important for maintaining normal nerve function and overall energy production, contributing to healthy growth and optimal metabolic function. Thiamin pyrophosphate (TPP), the active form of thiamin, is required for several enzymatic processes, making it a vital component of fish nutrition [41].

In the context of BSFLM, the reported vitamin B1 levels vary significantly, ranging from 0.24 mg/100 g, as found by Nyakeri et al. [30] to 1.98–2.0 mg/100 g, as observed by Zulkifli et al. [20]. This variability may be attributed to differences in the larvae's diet and processing methods, which affect their nutritional composition. For Nile tilapia, Lim et al. [35] recommended an optimal dietary inclusion level of 0.4 mg/100 g in tilapia feed, a level sufficient to support normal metabolic activities and prevent deficiency symptoms. Thiamin deficiency in fish can lead to reduced growth, loss of appetite, and neurological symptoms, as thiamin is critical for the functioning of enzymes involved in the citric acid cycle and the pentose phosphate pathway. Given the variability in thiamin content in BSFLM, it can be concluded that BSFLM could provide an adequate supply of vitamin B1 for Nile tilapia, especially when higher thiamin levels are present. However, in cases where BSFLM contains lower levels of thiamin, as reported by Nyakeri et al. [30], supplementation may be required to prevent deficiency.

#### 3.2.2. Vitamin B2 (Riboflavin)

Riboflavin (vitamin B2) is a crucial component of flavoproteins, which are involved in oxidation–reduction reactions in fish, playing a significant role in the metabolism of proteins, fats, and carbohydrates [42]. Riboflavin is necessary for the maintenance of healthy skin, eyes, and the overall metabolic function of Nile tilapia. Deficiency in riboflavin can lead to poor growth, cataracts, anorexia, fin erosion, and other physiological abnormalities, including short-body dwarfism and high mortality rates [43].

In BSFLM, riboflavin levels have been reported to range from 1.62 mg/100 g [20] to 24.74 mg/100 g [30]. This substantial variability could again be due to differences in the rearing conditions of the larvae and the substrates they are fed on. Regardless, these riboflavin levels in BSFLM are significantly higher than the Nile tilapia's requirement. According to Awad et al. [39], Nile tilapia requires only 1 mg/100 g of riboflavin in their diet. This suggests that BSFLM could be an excellent source of riboflavin, potentially exceeding the necessary dietary requirement for optimal growth and immune function in tilapia.

Although excess riboflavin is not harmful, its excess in the diet does not necessarily provide additional benefits once the fish's requirements are met. Therefore, while BSFLM can meet and exceed the riboflavin requirements of tilapia, attention should be paid to avoid unnecessary fortification of this vitamin in commercial feeds, which could increase feed costs without added nutritional benefit.

#### 3.2.3. Vitamin C (Ascorbic Acid)

Vitamin C, or ascorbic acid, is a powerful antioxidant and plays a vital role in collagen synthesis, tissue repair, and immune function in fish [44]. Unlike some animals, fish such as Nile tilapia are unable to synthesize vitamin C due to the lack of the enzyme L-gulonolactone oxidase, making it an essential dietary component [33]. Vitamin C deficiency in fish can result in poor wound healing, skeletal deformities, and compromised immune function, leading to increased susceptibility to infections.

The levels of vitamin C in BSFLM are relatively low, with reports ranging from 0.19 to 0.37 mg/100 g [20]. This is significantly below the required levels for Nile tilapia, which range from 5 to 42 mg/100 g depending on the size of the fish [33, 34]. Larger fish typically require higher levels of vitamin C, with 42 mg/100 g being recommended for fish weighing between 1.0 and 18.0 g. Given the instability of vitamin C during feed processing and storage, as noted by Kong et al. [45], much of the initial vitamin C content in BSFL-based feeds could be lost before the feed reaches the fish.

As such, it is clear that vitamin C supplementation would be necessary when using BSFLM as a primary feed ingredient. Practical strategies for supplementation include the addition of synthetic vitamin C (ascorbic acid) directly to the feed formulation, utilizing natural vitamin C-rich additives, and employing encapsulation techniques to protect vitamin C from degradation during feed processing and storage [46]. Additionally, optimizing feed processing conditions to minimize vitamin C loss and incorporating stabilized forms of vitamin C can enhance its retention and bioavailability in commercial feeds [47]. Although BSFLM can contribute to the overall nutrient profile, it cannot provide sufficient vitamin C on its own, especially in intensive aquaculture systems where fish are more reliant on formulated feeds than on natural food sources.

#### 3.2.4. Vitamin E

Vitamin E functions as a lipid-soluble antioxidant, protecting cell membranes, lipoproteins, and lipid stores from oxidative damage [48]. In Nile tilapia, vitamin E is crucial for maintaining the integrity of cellular structures and preventing oxidative stress, which can result in tissue damage and reduced immune function [49]. Vitamin E also plays a role in reproduction and is particularly important in diets that contain high levels of lipids, as it prevents the oxidation of polyunsaturated fatty acids (PUFAs).

The vitamin E content in BSFLM ranges from 0.62 to 1.30 mg/100 g [20], which is below the dietary requirement of Nile tilapia. Satoh et al. [50] recommend a dietary inclusion level of 5–10 mg/100 g for Nile tilapia fed a diet containing 5% lipid. For diets with higher lipid content (10%–15%), the requirement increases to 50 mg/100 g. Shiau and Huang [36] also suggest that tilapia requires ~4.2–4.4 mg/100 g of vitamin E for growth, with this amount increasing to 6–6.6 mg/100 g in diets with higher lipid levels. Given that the vitamin E content in BSFLM falls short of these requirements, additional supplementation would be necessary to meet the needs of tilapia, especially in diets containing significant amounts of lipids. Failure to meet the vitamin E requirement can lead to oxidative stress and reduced growth performance in fish.

#### 3.2.5. Pantothenic Acid (Vitamin B5)

Pantothenic acid, or vitamin B5, is integral to the synthesis of coenzyme A, which is essential for the metabolism of carbohydrates, fats, and proteins through the tricarboxylic acid (TCA) cycle [37]. In Nile tilapia, adequate levels of vitamin B5 are crucial for maintaining normal growth, energy production, and overall metabolic functions. Deficiency in pantothenic acid can lead to severe health issues, including poor growth rates, hemorrhaging, sluggishness, increased mortality, anemia, and hyperplasia of the gill epithelial cells [37]. To prevent such deficiency symptoms, a dietary inclusion level of 1 mg of calcium-pantothenate per 100 g of diet is recommended for Nile tilapia [37, 38]. BSFLM provides pantothenic acid at a concentration of 3.85 mg/100 g [31], which exceeds the nutritional requirements of tilapia. This indicates that incorporating BSFLM into Nile tilapia diets can effectively meet and potentially surpass the necessary levels of vitamin B5, thereby supporting optimal growth and metabolic health.

#### 3.2.6. Niacin (Vitamin B3)

Niacin, also known as vitamin B3, is a key component of coenzymes involved in energy metabolism. It is essential for the release of energy from carbohydrates, fats, and proteins [51]. In Nile tilapia, niacin is required for normal growth, skin health, and the prevention of metabolic disorders. Deficiency symptoms in fish include lesions, poor growth, and hemorrhages [32]. BSFLM contains 7.10 mg/100 g of niacin [20], which comfortably meets the Nile tilapia requirement of 2.6–12.1 mg/100 g [32]. This suggests that BSFLM could serve as an excellent source of niacin, reducing the need for additional supplementation in fish feed. Niacin deficiencies are relatively rare in fish that are fed balanced diets, and the niacin content in BSFLM is sufficient to prevent such deficiencies in Nile tilapia.

#### 3.2.7. Pyridoxine (Vitamin B6)

Pyridoxine, or vitamin B6, is involved in amino acid metabolism, acting as a coenzyme in the synthesis of neurotransmitters and hemoglobin. In fish, pyridoxine is crucial for protein utilization, growth, and the prevention of neurological disorders. Deficiency in pyridoxine can result in convulsions, poor growth, mouth lesions, and mortality [33]. The pyridoxine content in BSFLM is 0.60 mg/100 g [20], which falls within the Nile tilapia requirement range of 0.17–1.65 mg/100 g [33]. This indicates that BSFLM can provide an adequate amount of pyridoxine to support normal growth and metabolic function in Nile tilapia.

#### 3.2.8. Folic Acid (Vitamin B9)

Folic acid, or vitamin B9, serves as a precursor to the active coenzymes tetrahydrofolate, which are essential for one-carbon transfer reactions involved in amino acid and nucleotide metabolism [37]. These reactions are crucial for DNA synthesis, cell division, and overall growth. Deficiency in folic acid can result in impaired hematopoiesis, leading to anemia and reduced growth rates in fish [37]. BSFLM contains folic acid at levels of 0.27 mg/100 g [31], which significantly surpasses the dietary requirement of 0.05 mg/100 g for Nile tilapia [38]. This suggests that BSFLM is an excellent source of folic acid, ensuring that tilapia diets are well-supplied with this essential vitamin, thereby supporting healthy growth and efficient metabolic processes.

#### 3.2.9. Biotin (Vitamin B7)

Biotin, also known as vitamin B7, functions as a coenzyme for critical enzymes such as pyruvate carboxylase and acetyl-CoA carboxylase, which are involved in gluconeogenesis, fatty acid synthesis and the TCA cycle [37]. These enzymatic activities make biotin indispensable for the metabolism of amino acids, carbohydrates, and lipids in Nile tilapia, thereby supporting energy production and overall growth [37]. BSFLM contains biotin at a concentration of 0.035 mg/100 g [31], which exceeds the dietary requirement of 0.006 mg/100 g for Nile tilapia [38]. This indicates that BSFLM is a potent source of biotin, contributing significantly to meeting tilapia's nutritional needs and ensuring optimal metabolic health and growth performance.

#### 3.2.10. Vitamin B12 (Cobalamin)

Vitamin B12, or cobalamin, encompasses a group of cobalt-containing compounds, with cyanocobalamin being the biologically active form [37]. This vitamin is integral to the synthesis of nucleic acids and proteins, as well as the transfer of methyl groups in carbohydrate and fat metabolism. In Nile tilapia, vitamin B12-dependent enzymes are vital for these metabolic processes. However, tilapia can synthesize vitamin B12 in their gastrointestinal tract through bacterial synthesis, eliminating the need for dietary intake [52]. BSFLM contains vitamin B12 at a level of 0.00558 mg/100 g [31], but since Nile tilapia do not require dietary vitamin B12 for growth, the inclusion of BSFLM does not adversely affect tilapia nutrition regarding this vitamin. Thus, the presence of vitamin B12 in BSFLM is beneficial but not essential for Nile tilapia, providing an additional nutrient without necessitating supplementation.

#### 3.2.11. Choline

Choline is a vitamin-like nutrient essential for various biological functions in Nile tilapia, including the synthesis of phospholipids such as lecithin, which are critical components of cell membranes, and the production of acetylcholine, a neurotransmitter involved in muscle movement and cognitive function [37]. Additionally, choline provides labile methyl groups necessary for the synthesis of methylated metabolites, playing a role in metabolism and gene expression regulation. Studies have identified choline as essential for achieving maximum weight gain in fish, highlighting its importance in efficient growth and overall health [37]. BSFLM contains choline at a high concentration of 110 mg/100 g [31], which slightly exceeds the dietary requirement for Nile tilapia of 100 mg/100 g [38]. This suggests that BSFLM is an excellent source of choline, ensuring that tilapia diets meet their nutritional needs for optimal growth and metabolic function. The high choline content in BSFLM can contribute significantly to the overall lipid metabolism and neurological health of Nile tilapia, supporting robust growth and well-being.

As presented in Table 2, most of the vitamins in BSFLM exceed the dietary requirements for Nile tilapia, and it is crucial to manage these elevated levels to prevent potential negative impacts on fish health and to optimize feed efficiency. To avoid excessive fortification, several feed processing technologies can be employed. Precision formulation is essential, wherein feed manufacturers carefully balance the inclusion rates of BSFLM with other feed ingredients to ensure that vitamin levels remain within the optimal range for tilapia growth and health [53, 54]. Control of heat and light during feed processing is another critical factor, as excessive exposure can degrade sensitive vitamins such as riboflavin and thiamin, thereby naturally reducing their concentrations to more suitable levels [55]. Additionally, limiting synthetic additives and opting for natural feed components can help maintain the nutritional balance, preventing the inadvertent over-supplementation of certain vitamins [56]. Enzymatic treatments and microencapsulation techniques can also be utilized to modulate the availability and stability of vitamins in the feed, ensuring that they meet the specific needs of tilapia without surpassing the required amounts [57]. Furthermore, regular nutritional profiling and quality control measures should be integrated into the feed production process to continuously monitor and adjust vitamin levels as necessary.

### 3.3. Mineral Composition of BSFLM and Their Nutritional Requirements in Nile Tilapia

#### 3.3.1. Calcium

Calcium is the most prominent mineral in BSFLM, and its concentrations range from 13,005.12 to 26,515.88 mg/kg [20]. Calcium is critical for skeletal formation, muscle contraction, and other physiological functions in fish [28]. The optimal calcium requirement for Nile tilapia is around 3500–4200 mg/kg in diets for fish reared in water containing 27.1–33.3 mg Ca/L [58]. For tilapia larvae, calcium uptake from the water plays a significant role in growth, and the requirement is even higher at 13.27 ± 1.44 mg Ca^2+^/L [58]. The calcium content in BSFLM far exceeds the dietary requirements of Nile tilapia, suggesting that BSFLM can serve as an abundant source of calcium. However, it is important to consider that tilapia, like other fish, absorb calcium directly from the water in addition to dietary sources, which may reduce the necessity for such high calcium levels in the feed. The bioavailability of calcium from BSFLM also depends on the form in which it is present. Further research is needed to assess the bioavailability of calcium in BSFLM compared to traditional calcium sources, such as calcium carbonate, commonly used in aquaculture feeds.

#### 3.3.2. Phosphorus

Phosphorus is another critical mineral for fish, playing a key role in bone development, energy metabolism, and the formation of nucleic acids. The phosphorus content in BSFLM ranges from 6000 to 9100 mg/kg, closely aligning with the phosphorus requirement for Nile tilapia, which is around 9000 mg/kg [23]. Unlike calcium, phosphorus absorption from water is minimal, making dietary phosphorus a crucial component of tilapia feeds [59]. Phosphorus deficiency in fish diets can lead to reduced growth, poor feed conversion, and skeletal deformities. Studies have shown that phosphorus is one of the few minerals where supplementation in the diet has a direct impact on growth performance in Nile tilapia [60, 61]. Therefore, the phosphorus content in BSFLM is sufficient to meet the dietary needs of tilapia, which could reduce reliance on traditional phosphorus supplements, particularly in feed formulations that include plant-based ingredients with low phosphorus bioavailability.

#### 3.3.3. Potassium

Potassium levels in BSFLM range between 11,256.11 and 13,156.34 mg/kg [20]. This is significantly higher than the reported potassium requirement for Nile tilapia, which is approximately 2000–3000 mg/kg [23]. Potassium is essential for maintaining osmotic balance and acid–base regulation in fish. Although tilapia can absorb potassium from water, particularly in freshwater systems, dietary supplementation is often necessary in intensive aquaculture systems where mineral concentrations in the water are insufficient. The high potassium content in BSFLM may provide ample potassium to support these functions, but given the high concentrations, careful attention to feed formulation is required to avoid imbalances in mineral uptake that could affect the overall metabolic health of the fish.

#### 3.3.4. Sodium and Magnesium

Sodium and magnesium are present in BSFLM at concentrations of 3385.20–5028.42 mg/kg and 2505.98–3616.55 mg/kg, respectively [20]. While sodium plays a vital role in osmoregulation and nerve function [62], magnesium is involved in numerous enzymatic reactions and muscle function [63]. The optimal magnesium requirement for Nile tilapia is about 590 mg/kg [23], meaning the magnesium content in BSFLM exceeds this requirement. Adequate magnesium intake ensures proper muscle function and supports energy metabolism. However, excessive sodium and magnesium levels could lead to imbalances, especially when fish are raised in water with low mineral concentrations. Nevertheless, the naturally high magnesium content in BSFLM could be beneficial for reducing reliance on synthetic mineral premixes, particularly in low-intensity or organic aquaculture systems.

#### 3.3.5. Zinc (Zn)

Zn is a crucial trace element that supports a range of physiological processes in Nile tilapia, including growth, osmoregulation, immune function, bone and scale formation, and reproduction [64]. Zn also plays a significant role in metabolic pathways and is vital for biomineralization and the maintenance of innate immune functions in fish. Zn–gene interactions have been shown to positively influence growth performance [65]. Although tilapia can absorb Zn from water, this is not sufficient in controlled aquaculture systems, where dietary Zn supplementation is essential [66]. Numerous studies have demonstrated the benefits of Zn supplementation in tilapia diets. For example, an increase in Zn intake has been associated with improved weight gain and overall growth performance [67]. Conversely, Zn deficiency can result in significant health issues in tilapia, including reduced protein and carbohydrate digestibility, skin lesions, cataracts, stunted growth, and low serum Zn levels, ultimately leading to dwarfism [68]. It is also important to note that Zn bioavailability from feed ingredients can be influenced by the presence of tricalcium phosphate and phytates, both of which reduce the absorption of Zn [66].

Zn levels in BSFLM range from 303.07 to 1209.63 mg/kg [20], which is significantly higher than the dietary Zn requirements for Nile tilapia. Various studies suggest different optimum Zn levels for tilapia, with recommendations ranging from 30 to 79.51 mg/kg [66]. The consensus is that a minimum of 15–80 mg/kg is sufficient for tilapia species [69]. More specifically, Mjoun et al. [23] reported that Nile tilapia require between 3.1 and 22.1 mg/kg of dietary Zn.

Feeding tilapia diets containing very low levels of Zn (1–5 mg/kg) has been linked to poor growth and increased mortality, while diets supplemented with Zn levels above 30 mg/kg result in improved survival rates and growth [70]. However, excessive Zn intake, such as levels exceeding 327 mg/kg, has been associated with negative effects, including reduced growth, elevated blood glucose, cortisol concentrations, and increased levels of unionized ammonia [71]. Additionally, Zn concentrations above optimal levels can cause protein oxidation, contributing to the depletion of protein and lipid reserves in fish [72]. While the Zn content in BSFLM is considerably higher than the dietary requirements of Nile tilapia, it can still be used effectively in fish feeds, provided that care is taken to manage Zn levels to avoid toxicity. The optimum Zn requirement for Nile tilapia generally falls within the range of 30–80 mg/kg, with levels above this threshold potentially leading to adverse effects.

#### 3.3.6. Iron

Iron is a vital trace mineral present in BSFLM, with concentrations ranging from 300.75 to 689.24 mg/kg [20]. Iron plays a key role in hemoglobin synthesis and oxygen transportation in fish, which is crucial for maintaining metabolic functions and overall health. Iron deficiency in Nile tilapia has been linked to anemia, stunted growth, and compromised immune responses [73]. While tilapia primarily acquires iron through dietary intake, with minimal absorption through the gills [74], the bioavailability of iron from different dietary sources can vary significantly. Although iron is generally regarded as a nontoxic trace element, excessive dietary iron levels—such as 200 mg/kg—have been shown to pose toxicity risks to fish, potentially leading to mortality [75]. Studies suggest that the optimal dietary iron concentration for tilapia ranges between 30 and 170 mg/kg to prevent deficiency and promote growth [76]. Shiau and Su [77] also identified an iron requirement of 150–160 mg/kg for Nile tilapia, while dietary iron supplementation below 149 mg/kg may result in deficiency-related issues.

#### 3.3.7. Manganese (Mn)

Mn is an essential trace mineral that is widely distributed in both fish and animal tissues, where it plays critical roles in biochemical processes. One of its key functions is acting as an activator for metalloenzymes, including superoxide dismutase (SOD), which serves as a primary defense mechanism against oxidative stress. SOD exists in two forms depending on the metal it binds to: copper (Cu)–Zn SOD and Mn SOD. In particular, Mn SOD is vital for protecting cells from oxygen toxicity [78].

Mn is present in BSFLM at concentrations ranging from 134.93 to 162.51 mg/kg [20]. Although fish can absorb Mn from the surrounding water, dietary supplementation is often required in aquaculture systems to ensure that fish, such as Nile tilapia, receive sufficient amounts for optimal growth and health. The Mn content in BSFLM is relatively high, making it a potentially rich source of this mineral in fish diets. However, the inclusion of BSFLM in feeds may require monitoring to prevent potential mineral imbalances, especially when combined with other feed ingredients.

The Mn requirement for tilapia is still debated among researchers, with recommended dietary levels ranging from 13 to 15 mg/kg [79]. Studies have shown that Mn supplementation in tilapia diets increases Mn SOD activity, contributing to enhanced antioxidant defenses in the fish [80]. Additionally, higher levels of dietary Mn have been linked to increased Mn concentrations in the liver, bones, and muscles of Nile tilapia [80]. While Mn is necessary for tilapia health, excessive accumulation in fish tissues can pose risks to human consumers, as Mn can bioaccumulate through the food chain. The Mn content in BSFLM, while higher than the recommended requirement for tilapia, offers a viable source of minerals when used cautiously.

#### 3.3.8. Cu

Cu is a critical micronutrient for fish, playing essential roles in various biochemical and physiological processes. It supports mitochondrial respiration, oxidative reactions, DNA integrity, and transcription signaling, as well as serving as an allosteric factor for numerous enzymatic systems [81]. Additionally, Cu is vital for hematopoiesis, collagen synthesis, and oxidative phosphorylation, further emphasizing its importance in fish nutrition. Linder [82] noted that Cu functions as a cofactor for over 30 different enzymes, underscoring its integral role in cellular respiration and overall biochemical pathways. While fish can absorb Cu from both their diets and the surrounding water, freshwater species like tilapia have limited ability to meet their Cu requirements from the water alone, unlike their marine counterparts. Therefore, dietary supplementation of Cu is essential in freshwater aquaculture systems to prevent deficiencies [83]. Cu must be supplemented carefully within narrow ranges to balance nutritional needs and avoid toxicity, a task that is particularly challenging [83].

BSFLM contains Cu levels ranging from 13.69 to 29.10 mg/kg [20], which exceeds the dietary Cu requirements for tilapia. Research suggests that the optimal Cu requirement for various tilapia species ranges from 3 to 5 mg/kg [83], with a more specific requirement for juvenile tilapia estimated at 3.86 mg/kg based on broken-line analysis [84]. Cu deficiency (below 2 mg/kg) or excessive levels (above 56.34 mg/kg) can negatively impact tilapia, leading to reduced enzyme activity and potential toxicity [83]. Therefore, when using BSFLM as a feed ingredient, Cu levels should be carefully monitored to ensure they meet but do not exceed the optimal requirements for Nile tilapia.

Table 3 summarizes the mineral content of BSFLM and compares it to the dietary mineral requirements of Nile tilapia at different life stages.

In most of the cases, the mineral content of BSFLM exceeds the dietary requirements for Nile tilapia (Table 3), and therefore, it is essential to regulate these levels within formulated diets to prevent potential mineral imbalances and toxicity. This can be achieved through precision formulation, where feed manufacturers carefully calculate and adjust the inclusion rates of BSFLM based on the specific mineral needs of tilapia at different life stages [54]. Additionally, employing feed processing technologies such as defatting or mineral binding can help reduce the concentrations of excess minerals [85]. Blending BSFLM with other feed ingredients that are lower in specific minerals can also balance the overall mineral profile of the diet [86]. Furthermore, regular nutritional profiling and quality control measures are crucial to monitor and adjust mineral levels dynamically, ensuring that the formulated diets meet the precise nutritional requirements of Nile tilapia without exceeding safe thresholds.

### 3.4. Amino Acid Composition of BSFLM and Its Implications for Nile Tilapia Nutrition

The EAA profile of BSFLM (Table 4) highlights its potential as a protein source for Nile tilapia, given that amino acids are critical for fish growth, development, and overall health. EAAs, which must be supplied through the diet, are particularly important and should be considered when selecting a fish feed ingredient. The amino acid content in BSFLM is mainly influenced by the processing efficiency of the larvae. Defatting, which involves the mechanical or chemical removal of fat from the larvae, has been found to help increase the amino acid content of dried BSFLM [87]. Most studies have shown that the amino acid contents of the BSFLM do not differ much based on the culture substrate [88]. However, some EAAs, like methionine, have been reported to be relatively low in BSFLMs compared to FM. Generally, BSFLM has a good protein quality compared to FM and has higher contents of arginine, isoleucine, alanine, valine, histidine, and tryptophan [89].

One of the key EAAs is arginine, which plays a critical role in protein synthesis and growth in fish [90]. The arginine content in BSFLM ranges from 1.80% to 5.64% of dry matter (DM), based on the studies by Zulkifli et al. [20] and Huang et al. [21]. The optimal arginine requirement for Nile tilapia, according to Mjoun et al. [23], Diógenes et al. [91], and do Nascimento et al. [92], is between 1.36% and 4.20%. This means that BSFLM can meet or exceed the arginine needs of Nile tilapia, especially when using BSFLM with higher arginine concentrations. Sufficient arginine levels are particularly important for promoting rapid growth in tilapia fry and juveniles, where protein synthesis and growth rates are high [93]. However, depending on the specific source and processing of the larvae, some formulations may require additional supplementation to consistently meet this requirement across different batches of BSFLM.

Histidine, another EAA, is necessary for muscle development and the biosynthesis of hemoglobin [94]. The histidine content in BSFLM ranges between 1.18% and 4.63%, well above the 0.47%–1.72% required by Nile tilapia [23, 91, 92]. This suggests that BSFLM is an excellent source of histidine, capable of more than meeting the dietary needs of tilapia across all life stages. Adequate histidine levels in the diet can support better growth rates and improved feed conversion efficiency, particularly in juvenile tilapia, which have a higher demand for EAAs for tissue development. The leucine content of BSFLM is particularly variable, ranging from 2.52% to 8.80% DM [21]. Nile tilapia require approximately 1.33%–3.39% leucine in their diet [23], which means that BSFLM can supply ample leucine, particularly when larvae with higher concentrations are used. Leucine is important for protein metabolism and muscle repair, making it crucial for maintaining healthy growth in fish. Its presence in high amounts in BSFLM means that this alternative feed ingredient could effectively support tissue repair and growth, especially in fast-growing fish, which are common in aquaculture systems aimed at optimizing production.

Similarly, lysine is a critical amino acid for tilapia, particularly because it influences growth rates, feed efficiency, and nitrogen retention [95]. Lysine content in BSFLM ranges from 1.91% to 7.03% DM, compared to the 1.56%–5.12% lysine requirement for Nile tilapia (Table 4). Lysine is often the limiting amino acid in fish diets, meaning that if its levels are insufficient, growth performance can be impaired even if other nutrients are adequate. The higher end of the lysine range in BSFLM meets or exceeds the requirement for tilapia, making BSFLM a potentially valuable lysine source. However, supplementation might be necessary for larvae with lower lysine content to avoid any growth restrictions due to lysine deficiency, especially in high-intensity production systems where maximizing growth is critical.

Methionine, another limiting amino acid in many fish diets, is found in concentrations ranging from 0.53% to 2.74% in BSFLM tilapia [20, 21], while the optimal requirement for Nile tilapia is 0.65%–2.68% [23]. Methionine is essential for protein synthesis, as well as for supporting antioxidant activity and immune function in fish [96]. The lower range of methionine content in BSFLM might present a challenge, particularly in intensive aquaculture systems, where even slight deficiencies can impact fish growth and health. Therefore, while BSFLM can contribute to the methionine requirements, it may not always be sufficient on its own, necessitating the inclusion of other methionine-rich ingredients or synthetic methionine in the feed formulation. Phenylalanine and threonine are also important amino acids for fish growth and immune system support [97]. The phenylalanine content in BSFLM ranges from 1.35% to 6.24%, compared to the 0.01%–3.75% required by Nile tilapia [23]. Threonine, on the other hand, ranges from 1.42% to 4.48% [20], also with a requirement of 3.75%. The presence of these amino acids in BSFLM at levels close to or exceeding the tilapia requirements makes this insect-based feed a promising source for both phenylalanine and threonine. Adequate levels of these amino acids in the diet can improve protein metabolism and immune function, which are critical for maintaining fish health, especially in stressful farming conditions such as high stocking densities. Valine, an EAA involved in muscle growth and repair, is found in concentrations ranging from 2.29% to 5.80% in BSFLM, compared to the 0.96%–2.80% requirement for Nile tilapia [23]. The valine content in BSFLM suggests that it is more than capable of meeting the tilapia's dietary requirements. This contributes to the overall potential of BSFLM to support muscle development and efficient protein utilization in tilapia.

Therefore, the amino acid profile of BSFLM aligns closely with the nutritional requirements of Nile tilapia, especially for key amino acids such as histidine, lysine, leucine, and valine. While BSFLM can meet or exceed the requirements for most EAAs, there are some areas, particularly with methionine and isoleucine, where supplementation might be needed. Nonetheless, the high variability in the amino acid composition of BSFLM, which can be influenced by rearing and processing conditions, indicates that optimizing BSFLM production to consistently meet the nutritional needs of tilapia is crucial. Further research into standardizing BSFLM production methods, as well as investigating the use of processed BSFLM in combination with other protein sources, can help overcome any amino acid deficiencies and enhance the overall viability of this alternative feed ingredient in Nile tilapia aquaculture systems.

### 3.5. Fatty Acid Composition of BSFLM and Its Implications for Nile Tilapia Nutrition

The fatty acid composition of BSFLM plays a pivotal role in its potential as a sustainable feed ingredient for Nile tilapia, especially considering the essential fatty acid (EFA) requirements of this species. Nile tilapia, like many freshwater fish, cannot synthesize certain PUFAs such as linoleic acid (18:2n-6) and alpha-linolenic acid (18:3n-3), making them essential components that must be supplied through the diet [98]. The review of the fatty acid composition of BSFLM shows a diverse profile of saturated fatty acids (SFAs), monounsaturated fatty acids (MUFAs), and PUFAs, each contributing differently to the overall health and growth of Nile tilapia.

One of the striking aspects of BSFLM is its high content of SFAs, which comprise between 46.69% and 77.47% of total fat, with lauric acid (12:0) being the dominant SFA, ranging from 17.89 to 37.18% [20]. While SFAs do not serve the same critical biological functions as PUFAs, they do provide a stable energy source. Lauric acid has been shown to possess antimicrobial properties, which could contribute to improved fish health by enhancing resistance to pathogens [99]. However, the high SFA content, especially of lauric acid, might need careful management in formulating feeds, as excessive SFAs can impact lipid metabolism and affect the overall fatty acid profile of tilapia. Furthermore, the relatively high levels of other SFAs, such as palmitic acid (16:0) and myristic acid (14:0), which range from 20.65 to 24.59% and 5.21%–11.77%, respectively, suggesting that BSFLM can provide substantial energy and specific fatty acid needs related to membrane fluidity and immune function of the fish, as mirrored in PUFAs levels [100].

In contrast to the SFAs, the MUFAs in BSFLM, such as oleic acid (18:1—cis n9), account for 9.28%–15.35% of total fat [20]. Oleic acid plays a significant role in maintaining cell membrane integrity and regulating lipid metabolism in fish [101]. The presence of MUFAs in moderate amounts provides a balance between energy provision and metabolic health, making them beneficial components in Nile tilapia diets. However, the sum of MUFAs in BSFLM is relatively low, ranging from 11.03%–18.02%, indicating that while BSFLM can contribute to the tilapia's MUFA needs, it may not be sufficient as the sole lipid source for optimal growth and health [20]. Supplementing BSFLM-based feeds with other MUFA-rich sources might be necessary to ensure a balanced fatty acid profile in Nile tilapia.

The most critical aspect of the BSFLM fatty acid composition, especially in relation to tilapia nutrition, lies in its PUFAs, particularly the omega-3 (n-3) and omega-6 (n-6) fatty acids. BSFLM contains both alpha-linolenic acid (18:3n-3) and linoleic acid (18:2n-6), the two essential PUFAs that Nile tilapia requires. The alpha-linolenic acid content in BSFLM ranges from 0.32% to 1.99%, while linoleic acid ranges from 4.71% to 24.08% [20]. Research has shown that freshwater fish like Nile tilapia can elongate and desaturate linoleic acid to arachidonic acid (20:4n-6) and alpha-linolenic acid to eicosapentaenoic acid (EPA, 20:5n-3) and docosahexaenoic acid (DHA, 22:6n-3), making them less dependent on long-chain PUFAs compared to marine fish [102]. This means that the presence of linoleic acid and alpha-linolenic acid in BSFLM could meet the EFA needs of Nile tilapia, supporting growth, immune function, and overall health.

However, the balance between omega-3 and omega-6 fatty acids is crucial. The omega-3 to omega-6 ratio in BSFLM is notably low, ranging from 0.05 to 0.12, reflecting a much higher concentration of omega-6 fatty acids, particularly linoleic acid, compared to omega-3 fatty acids [20]. The optimum dietary requirement for omega-6 fatty acids in Nile tilapia is estimated to be around 0.5% of the diet [103], and the high levels of linoleic acid in BSFLM, especially at the upper range of 24.08%, suggest that it could easily meet or exceed this requirement. However, an excess of omega-6 fatty acids relative to omega-3 can lead to an imbalance in the production of eicosanoids, which are signaling molecules involved in inflammatory responses [104]. This imbalance can potentially have negative effects on fish health, particularly in intensive aquaculture systems where fish are exposed to higher stress levels.

The low levels of omega-3 fatty acids in BSFLM, particularly alpha-linolenic acid, could limit its ability to fully meet the EFA requirements for optimal tilapia health. While tilapia can convert C18 fatty acids like alpha-linolenic acid into longer-chain n-3 fatty acids such as EPA and DHA, the efficiency of this conversion can be limited under certain farming conditions, especially in fast-growing fish. Therefore, while BSFLM provides essential n-3 and n-6 fatty acids, its use as a primary lipid source in tilapia diets may require supplementation with other ingredients rich in omega-3 fatty acids [105], such as fish oil or algae-based oils, to ensure an optimal balance between n-3 and n-6 fatty acids and to promote anti-inflammatory responses and membrane fluidity.

### 3.6. Possible Solutions to Excess Lipid Content in BSFLM-Based Diets

The high lipid content of BSFLM presents both opportunities and challenges in formulating balanced diets for Nile tilapia. While lipids are essential for energy provision, cell membrane integrity, and the absorption of fat-soluble vitamins, excessive lipid levels can lead to issues such as poor FCRs, fat deposition, and oxidative stress in fish [106]. Moreover, the substantial lipid content in BSFLM contributes to elevated levels of SFAs, which necessitates careful management to maintain optimal fatty acid profiles in tilapia diets. Effective defatting of BSFLM is therefore crucial to tailor its lipid content to the specific nutritional requirements of Nile tilapia, ensuring optimal growth performance and health [107]. Various advanced feed processing technologies have been developed and refined to address the challenge of lipid removal from BSFLM, each with its unique advantages and limitations.

Solvent extraction remains one of the most widely utilized methods for defatting due to its high efficiency and scalability [108]. This technique involves the use of organic solvents such as hexane, ethanol, or a mixture of solvents to dissolve and extract lipids from BSFLM [109]. The process typically includes mixing BSFLM with the solvent, allowing the lipids to dissolve, followed by the separation of the solvent–lipid mixture from the defatted meal through filtration or centrifugation [108]. The solvent is then evaporated, leaving behind the defatted BSFLM. Solvent extraction is highly efficient, often achieving lipid removal rates exceeding 90%, and is easily scalable for commercial operations [110]. However, the method has significant drawbacks, including the potential for solvent residues in the defatted meal, which pose health risks to fish and consumers. Additionally, the environmental impact of solvent disposal necessitates careful management to prevent contamination. Recent advancements focus on optimizing solvent types and extraction conditions to minimize residue levels and environmental footprint, such as employing food-grade solvents like ethanol and implementing closed-loop systems to enhance solvent recovery and reuse [111].

Mechanical pressing, including expeller pressing and screw pressing, offers a solvent-free alternative for lipid removal [112]. This physical method leverages mechanical force to extract lipids from BSFLM by subjecting the meal to high pressure and temperature, causing the lipids to be expelled [27]. Screw pressing utilizes a screw mechanism to apply continuous pressure, facilitating the separation of lipids from the meal [113]. Mechanical pressing is advantageous as it eliminates the need for organic solvents, resulting in cleaner, defatted meals with no chemical residues [112]. Additionally, the lower temperatures used in some mechanical pressing methods help preserve heat-sensitive vitamins and amino acids, maintaining the nutritional integrity of the defatted meal [114]. However, mechanical pressing typically achieves lower lipid removal rates compared to solvent extraction, often around 70%–80% [115]. The high initial investment required for specialized pressing equipment and the potential for equipment wear and maintenance costs are notable disadvantages. To enhance efficiency, mechanical pressing is often combined with other defatting techniques, such as enzymatic treatments, to improve lipid extraction while preserving nutrient quality [116].

Supercritical fluid extraction (SFE) represents a cutting-edge technology that employs supercritical CO_2_ as a solvent under high pressure and temperature conditions to extract lipids from BSFLM [117]. SFE offers a green alternative to traditional solvent extraction, as CO_2_ is nontoxic, nonflammable, and easily removed from the final product. This method is highly selective, capable of targeting specific lipid fractions and allowing for the tailoring of fatty acid profiles to meet the nutritional needs of Nile tilapia. Furthermore, SFE leaves no solvent residues, ensuring the safety and quality of the defatted meal [118]. Despite its advantages, SFE is associated with high operational costs due to the specialized equipment and significant energy input required, making it less economically feasible for some operations. Additionally, the technical complexity of optimizing extraction parameters to maximize efficiency while preserving essential nutrients necessitates substantial expertise [119]. Nevertheless, SFE holds promise for producing high-quality defatted BSFLM with minimal environmental impact, especially when advancements continue to reduce costs and improve process efficiency.

Freeze/thawing is a method that involves repeatedly freezing and thawing BSFLM to disrupt cellular structures and facilitate lipid release and subsequent removal [120]. This technique leverages the expansion of ice crystals during freezing to rupture cellular membranes, making lipids more accessible for extraction [121]. The primary advantage of freeze/thawing is that it is a nondestructive process that preserves the structural integrity and nutrient quality of the defatted meal. Additionally, it avoids the use of chemical solvents, thereby eliminating residue concerns [122]. However, freeze/thawing is energy-intensive, requiring significant energy input for the freezing and thawing cycles, which can increase operational costs. Moreover, the method typically achieves lower lipid removal rates, around 50%–60%, necessitating the use of additional defatting steps to achieve desired lipid levels [120]. Combining freeze/thawing with other defatting techniques, such as centrifugation or mechanical pressing, can enhance overall lipid removal efficiency while maintaining the nutritional quality of BSFLM [123].

Integrated approaches that combine multiple defatting technologies can synergistically improve lipid removal efficiency and optimize the nutritional profile of BSFLM. For instance, mechanical pressing followed by solvent extraction can achieve higher lipid removal rates while minimizing solvent usage and preserving nutrient quality [121]. Similarly, integrating enzymatic treatments with mechanical pressing can enhance lipid release and separation, resulting in more efficient defatting processes [116]. These hybrid methods leverage the strengths of each individual technique, addressing their respective limitations to produce defatted BSFLM that meets the specific nutritional requirements of Nile tilapia without compromising feed quality.

## 4. Growth Performance of Nile Tilapia in Various Culture Systems

Table 5 provides growth performance parameters of Nile tilapia reared in various aquaculture systems and fed diets with different levels of FM replacement by BSFLM. It is essential to acknowledge that the data compiled in this table originate from diverse independent studies conducted across a range of aquaculture systems, each with varying environmental conditions and husbandry practices. Due to differences in experimental methodologies, culture conditions, and inclusion rates of BSFLM, direct comparisons across all studies are limited and should be interpreted with caution. Nonetheless, despite these variations, the collective findings consistently demonstrate that BSFLM can effectively replace traditional protein sources, thereby enhancing the sustainability and economic viability of Nile tilapia production in diverse aquaculture systems.

### 4.1. SGR of Nile Tilapia

The SGR is crucial in assessing the growth efficiency of Nile tilapia across different aquaculture systems, providing insight into how well the fish convert feed into biomass [131]. The comparison of SGR across various studies reveals how different aquaculture systems, in conjunction with other factors like FM replacement with BSFLM, affect the growth performance of tilapia. The hapas set in the ponds system consistently demonstrated significant variations in SGR depending on culture duration and FM replacement levels. For instance, in the study by Rana et al. [124], which used a short 28-day culture period and a 50% FM replacement, an exceptionally high SGR of 3.743 ± 0.35 was achieved. This high growth rate can be attributed to several factors. The initial small fish size (0.99 ± 0.17 g) placed the fish in the early juvenile stage, where growth rates are typically exponential due to higher metabolic efficiency and nutrient assimilation. Additionally, the controlled yet semi-natural pond environment, where the fish had access to both formulated feed and natural food sources, may have further enhanced nutrient availability, thereby optimizing growth. The 50% FM replacement provided a balanced protein source, likely ensuring sufficient EAAs for protein synthesis, while the short culture period minimized the exposure of fish to environmental fluctuations and stressors.

In contrast, the same hapas set in the ponds system used in Ouko et al. [125] yielded a much lower SGR of 1.31 ± 0.03 despite utilizing the same 50% FM replacement level. The key factor driving this discrepancy in SGR is the longer culture period of 147 days. As fish age, their growth naturally decelerates, particularly as they approach sexual maturity [132], which reduces the efficiency of nutrient conversion into biomass. The smaller initial fish size (0.11 ± 0.01 g) may have promoted rapid early growth, but the extended culture period likely introduced additional environmental stressors, such as seasonal variations in water temperature, dissolved oxygen, and other water quality parameters, all of which could have negatively affected growth performance. Moreover, while the FCR was slightly better in Ouko et al. [125], the extended duration and environmental changes outweighed the benefits of efficient feed use, resulting in the lower SGR.

Nairuti et al. [126] also employed the hapas set in the ponds system but with a complete replacement of FM with BSFLM over a 72-day period, reporting an SGR of 1.1 ± 0.02—a value lower than those observed with partial replacements [124, 125]. This reduction in growth rate may be influenced by multiple factors beyond just the absence of FM: the significantly larger initial fish size (52.2 ± 0.74 g) naturally limits relative growth potential, and the complete dietary shift to BSFLM can subtly alter the amino acid and fatty acid profiles, potentially lowering growth efficiency in comparison to partial replacements. Although the semi-natural pond environment likely provided some nutritional supplementation via natural feed organisms, the lower SGR underscores that 100% FM replacement with BSFLM may require careful formulation adjustments or supplementation to achieve growth rates comparable to those seen at 50% or 75% replacement levels.

In a different system, cages set in earthen ponds, the SGR was significantly lower. Mathai et al. [127] reported an SGR of 0.52 ± 0.02 over an extended culture period of 182 days with a 75% FM replacement. The lower SGR can be attributed to the constraints of the cage system, which limits water exchange and space, potentially leading to competition for feed and reduced growth rates. Additionally, the longer culture period, which allowed the fish to reach a size where growth naturally slows, combined with the higher FM replacement level, may have introduced nutritional imbalances that further constrained growth. The relatively high FCR of 2.13 ± 0.06 in this system reinforces the observation of less efficient feed conversion and slower growth. In contrast, Munguti et al. [19], which used the same cage system but with a lower 50% FM replacement, reported a marginally higher SGR of 0.60 ± 0.02. The improved growth performance in this study can be linked to the more balanced nutritional profile provided by the 50% FM replacement, which likely offered better amino acid and lipid availability compared to the higher replacement level used in Mathai et al. [127]. However, the inherent limitations of the cage system, including restricted water flow and environmental enrichment, still resulted in relatively low SGR values despite the moderate improvement in feed efficiency (FCR of 1.46 ± 0.16).

Moving to a more controlled system, Fayed et al. [128] reported an SGR of 0.82 ± 0.001 in glass tanks with a 25% FM replacement over 84 days. The glass tank system provides optimal control over water quality and temperature, likely contributing to consistent growth but still resulting in a relatively low SGR. This may be partly due to the artificial environment limiting natural feeding behavior and activity levels, both of which are important for stimulating growth. Although the low FM replacement level ensured a nutritionally complete diet, the lack of environmental enrichment and restricted space likely curbed potential growth improvements. Similarly, under 100% FM replacement conditions [18], the SGR (1.30 ± 0.17) remained lower than those observed with partial replacements, suggesting that while BSFLM can support growth in controlled environments, the absence of FM-derived nutrients may require careful supplementation or formulation adjustments to achieve higher growth efficiencies.

When examining the results from Nairuti et al. [126] and Tippayadara et al. [18], both of which employed 100% FM replacement with BSFLM, the relatively low SGR values (1.1 ± 0.02 and 1.30 ± 0.17, respectively) stand in contrast to higher SGRs observed in studies utilizing partial FM substitutions. However, these differences should be interpreted with caution since each study was conducted under distinct initial fish weights, culture durations, and environmental conditions. Numerous factors—ranging from the nature of rearing environments, stocking densities, and feeding strategies to the inherent physiological differences related to fish size—can influence growth performance. Consequently, the lower SGRs reported at full FM replacement may not be solely attributed to the nutritional adequacy of BSFLM. Instead, these results highlight the complexity of drawing direct comparisons across studies and emphasize the need for future research to control and standardize variables when evaluating different levels of FM replacement.

In plastic tanks, Muin et al. [130] achieved a significantly higher SGR of 2.43 ± 0.04 over a short 56-day period with a 50% FM replacement. The high SGR in this system can be attributed to the controlled conditions of the plastic tanks, where water quality and feeding regimes were tightly managed, reducing environmental stressors and promoting rapid growth, especially in juvenile fish. The short culture period, combined with the balanced diet provided by the 50% FM replacement, allowed the fish to grow rapidly before reaching the size where growth naturally decelerates. However, the higher FCR of 2.91 ± 0.10 in this system suggests that while growth was rapid, feed conversion was less efficient compared to other systems.

Based on the SGR analysis across different aquaculture systems, hapas set in ponds with shorter culture periods, as observed in Rana et al. [124], demonstrated the best overall growth performance, with the highest SGR of 3.743 ± 0.35. This system is ideal for rapid early-stage growth, particularly when a 50% FM replacement level is used. For longer culture periods, plastic tanks, as seen in Muin et al. [130], offer the next best alternative, achieving an SGR of 2.43 ± 0.04. However, for farmers seeking a balance between long-term growth and sustainability, hapas in earthen ponds, particularly those using 100% BSFL, provide a viable option, supporting substantial growth while maintaining good feed efficiency and survival rates. Therefore, based on SGR, hapas set in ponds for short-term culture, followed by plastic tanks for controlled environments, offer the most efficient growth outcomes for Nile tilapia fed with BSFL.

### 4.2. FCR of Nile Tilapia

The FCR is a fundamental metric in aquaculture, representing the efficiency with which feed is converted into biomass [133]. A lower FCR indicates higher feed efficiency, meaning less feed is required to produce a unit of fish weight gain. In aquaculture systems, FCR can be influenced by several factors, including FM replacement levels, environmental conditions, culture period, stocking density, feed composition, and fish health. In hapas set in ponds, Rana et al. [124] reported an FCR of 1.7 ± 0.2 over a 28-day culture period, with a 50% FM replacement using BSFL. This relatively low FCR reflects a high level of feed efficiency, likely due to the short culture period and the small initial fish size (0.99 ± 0.17 g), which allowed for rapid growth with minimal feed wastage. Fish at early life stages tend to convert feed into biomass more efficiently [134], especially when the diet provides a balanced nutritional profile. The controlled environment of hapas within a pond system may have contributed to minimizing feed losses, ensuring that most of the feed was consumed rather than being lost to the environment. The pond environment could also have provided natural food sources, which may have supplemented the BSF-based diet, contributing to the relatively low FCR.

In a longer-term study using hapas set in ponds, Ouko et al. [125] reported a slightly lower FCR of 1.57 ± 0.02 with the same 50% FM replacement over a 147-day culture period. The lower FCR compared to Rana et al. [124] suggests more efficient feed utilization over the extended period, possibly due to the fish's adaptation to the BSF-based diet. However, the extended culture period might have allowed the fish to better utilize the nutrients from the feed as their digestive systems matured. Additionally, the smaller initial fish size (0.11 ± 0.01 g) meant that the fish were in a phase of rapid growth for a longer period, which could have contributed to the better FCR. The hapas system likely minimized competition for feed, allowing each fish to access sufficient nutrients, further enhancing feed efficiency.

In a study by Nairuti et al. [126] conducted in hapas nets in an earthen pond, the FCR was 1.1 ± 0.01, the lowest among all the systems reviewed. This study used a 100% FM replacement with BSFL over a 72-day culture period, starting with an initial fish weight of 52.2 ± 0.74 g. The low FCR in this system indicates exceptionally efficient feed utilization despite the complete replacement of FM. Several factors could explain this outcome. The earthen pond environment likely provided natural feed sources, such as algae and zooplankton, which could have supplemented the BSF diet. Second, the hapas nets may have helped reduce feed wastage by containing the feed within a confined space, making it easier for the fish to consume it. Additionally, the larger initial fish size means the fish were at a more advanced developmental stage, where nutrient absorption and feed conversion are more efficient. The high survival rate (98.3% ± 1.67%) also suggests that the fish were healthy, which is critical for maintaining low FCRs, as unhealthy fish tend to convert feed less efficiently.

In cages built in an earthen pond, Mathai et al. [127] reported a higher FCR of 2.13 ± 0.06 over an 182-day culture period with a 75% FM replacement. The higher FCR in this system indicates less efficient feed utilization compared to the other studies. The cage system, while allowing water flow and exchange, may have led to more feed being lost to the environment compared to the more contained hapas systems. Additionally, the extended culture period likely reduced the efficiency of feed conversion as the fish grew larger and reached a stage where growth slowed down, but feed intake remained high. The higher FM replacement level might also have impacted the digestibility of the diet, as BSFL, while rich in protein, may lack some of the EAAs and lipids that FM provides, affecting long-term growth and feed efficiency.

Munguti et al. [19], who also studied fish in cages in an earthen pond, reported a lower FCR of 1.46 ± 0.16 over the same 182-day culture period with a 50% FM replacement. The lower FCR compared to Mathai et al. [127] suggests that the 50% replacement level provided a more balanced diet, allowing for more efficient feed conversion. Additionally, the initial fish weight was smaller (24.72 ± 0.39 g) in Munguti et al. [19] compared to Mathai et al. [127] (33.88 ± 2.18), which may have enabled the fish in the former study to utilize feed more efficiently during the initial growth phase, contributing to the observed lower FCR. The extended culture period, however, still contributed to the overall higher FCR compared to shorter-term studies, as the fish likely reached a growth plateau where additional feed intake was not fully converted into biomass.

In glass aquaria, Fayed et al. [128] reported an FCR of 1.22 ± 0.005 over an 84-day culture period with a 25% FM replacement. The low FCR in this study reflects high feed efficiency, which can be attributed to the controlled environment of the glass aquaria. In such systems, water quality, temperature, and feeding can be closely monitored, reducing feed wastage and ensuring that the fish have optimal conditions for growth. The low FM replacement level likely provided a nutritionally complete diet, allowing the fish to efficiently convert feed into biomass. Additionally, the relatively small initial fish size (4.25 ± 0.08 g) suggests that the fish were still in a phase of rapid growth, which is typically associated with lower FCRs.

In glass tanks, Tippayadara et al. [18] observed a higher FCR of 2.23 ± 0.15 over an 84-day culture period with a 100% FM replacement. The higher FCR in this study could be explained by the complete replacement of FM, which may have resulted in nutritional deficiencies, particularly in EAAs, fatty acids, or minerals that are more abundant in FM. While BSFL provides a high-protein alternative, their nutrient profile may not be as optimal for long-term growth in a controlled environment like glass tanks, where fish rely entirely on the provided feed. Additionally, the initial fish weight of 14.51 ± 2.06 g indicates that the fish were past the juvenile stage, where feed conversion typically starts to become less efficient.

In plastic tanks, Muin et al. [130] reported an FCR of 2.91 ± 0.10, the highest among the studies reviewed, over a 56-day culture period with a 50% FM replacement. The high FCR suggests low feed efficiency, which could be attributed to several factors. The initial fish weight of 3.03 ± 0.05 g indicates that the fish were very small at the start of the study, which might have led to high feed wastage if the feeding strategies were not well adapted to the small size of the fish. Additionally, the short culture period may not have allowed the fish to fully adapt to the BSF-based diet, particularly if the feed was not optimized for small, early-stage tilapia. The controlled environment of plastic tanks, while useful for maintaining water quality, may have limited the natural feeding behaviors of the fish, further contributing to the high FCR.

Limbu et al. [129], also using plastic tanks, reported a much lower FCR of 0.89 ± 0.02 over 84 days with a 75% FM replacement. The lower FCR in this study indicates highly efficient feed conversion, which may be attributed to the controlled conditions of the plastic tanks, combined with a diet that was likely well-balanced despite the high level of FM replacement. The small initial fish weight (0.001 g) suggests that the fish were in the early stages of growth, where feed conversion is typically more efficient. The extended culture period allowed the fish to grow steadily, maximizing feed efficiency as they transitioned from early juvenile stages to more mature phases.

Based on the FCR analysis, hapas nets in earthen ponds with a 100% FM replacement reported by Nairuti et al. [126] achieved the best feed efficiency, with the lowest FCR of 1.1 ± 0.01. This system benefited from a combination of natural feed supplementation in the earthen pond environment and the efficient containment of feed within the hapas. For long-term culture, hapas set in ponds, as seen in Ouko et al. [125], also demonstrated good feed efficiency with a lower FCR of 1.57 ± 0.02 despite the extended culture period. Plastic tanks, as reported by Limbu et al. [129], also showed highly efficient feed conversion, making them a viable option for controlled environments with a high FM replacement level. Therefore, for optimal FCR, hapas systems in earthen ponds with high FM replacement levels offer the most efficient growth outcomes, while plastic tanks can be considered a strong alternative in controlled, intensive aquaculture setups.

### 4.3. Survival Rate

The survival rate is a critical measure in aquaculture, reflecting the health and adaptability of fish to specific environmental conditions, feed quality, and management practices [135]. Across the various studies comparing Nile tilapia culture systems with different levels of BSFL as FM replacements, the survival rate reveals how well the fish respond to these conditions. In hapas set in ponds, the survival rates were generally high, though differences emerged based on the culture period and the FM replacement level. Rana et al. [124], with a 50% FM replacement over a short 28-day period, achieved a strong survival rate of 93.33%. The short duration likely minimized the fish's exposure to stressors such as disease outbreaks or water quality fluctuations, which often affect longer-term studies. Additionally, the semi-natural pond environment provided both controlled feeding and access to natural food sources, supporting the fish's nutritional needs and promoting health.

In a much longer study, Ouko et al. [125], which also used 50% FM replacement over 147 days, saw an even higher survival rate of 96.74% ± 3.18%. The extended culture period allowed the fish more time to adapt to the BSF-based diet, and the stable pond environment likely provided a continuous supply of natural nutrients, supplementing the formulated feed. This combination of a balanced diet and natural enrichment supported high survival, suggesting that longer exposure to the semi-controlled pond system, along with gradual dietary adaptation, can enhance the overall resilience of the fish.

In the case of Nairuti et al. [126], where 100% FM replacement was employed in hapas nets within an earthen pond, the survival rate was the highest among the pond-based systems, at 98.3% ± 1.67%. Despite the complete replacement of FM, the earthen pond's natural ecosystem likely provided critical supplemental nutrients such as algae and zooplankton. These natural food sources may have filled any nutritional gaps that could arise from the BSF-based feed, ensuring that the fish received a well-rounded diet. Additionally, the hapas nets reduced environmental stress by offering protection from predators, allowing the fish to focus on growth and development without the need to expend energy on survival.

Moving to cage systems in earthen ponds, the survival rates remained robust, even though these systems are more exposed to environmental fluctuations than hapas. Both Mathai et al. [127] and Kariuki et al. [86], despite using different FM replacement levels, reported similarly high survival outcomes. Mathai et al., using a 75% FM replacement over 182 days, reported a survival rate of 95.83% ± 3.31%, while Kariuki et al. [86], with 50% FM replacement over the same period, achieved a perfect survival rate of 100%. The slight difference in survival rates between these two studies may be linked to the FM replacement levels. The lower replacement level in Kariuki et al. [86] may have provided a more nutritionally complete diet, better supporting the fish's health and stress resistance. Nonetheless, both studies underscore the resilience of Nile tilapia in cage systems, which, despite being more exposed to environmental fluctuations, still benefit from natural water exchange and nutrient flow in the earthen pond.

Glass tanks and aquaria, which offer the most controlled environments, also supported high survival rates. In Fayed et al. [128], where a 25% FM replacement was used in glass aquaria, the survival rate was 98.00% ± 0.58%. The controlled conditions in aquaria—where water quality, temperature, and feeding are closely monitored—eliminated many of the risks associated with environmental fluctuations or disease outbreaks. The low FM replacement level likely provided a nutritionally rich diet, contributing to the observed high survival rate. Similarly, Tippayadara et al. [18], using 100% FM replacement in glass tanks, also achieved a perfect survival rate of 100%, suggesting that even with complete FM replacement, a tightly controlled environment can compensate for any potential nutritional deficiencies by maintaining optimal water quality and feeding regimes.

Plastic tanks presented a varied but generally favorable performance in terms of survival. In Limbu et al. [129], the use of a 75% FM replacement yielded a high survival rate of 96.46% ± 1.61%, indicating that even with higher replacement levels, the controlled conditions of plastic tanks—where water quality is consistently managed—can support strong fish health. The ability to closely monitor the environment and prevent disease outbreaks likely contributed to this high survival outcome. Similarly, Muin et al. [130], with 50% FM replacement in plastic tanks, achieved a perfect survival rate of 100%. This demonstrates that plastic tanks, when combined with a balanced diet and effective management practices, can be highly effective in maintaining fish health over shorter culture periods.

When comparing these systems, cages built in earthen ponds with 50% FM replacement, as seen in Kariuki et al. [86] and Munguti et al. [19], stand out as the top performers in terms of survival. Both studies achieved a 100% survival rate over long 182-day culture periods. This suggests that the cage system, combined with the benefits of natural environmental enrichment and a balanced diet, creates an ideal environment for supporting fish health. The cage system within an earthen pond allows for natural water exchange and the presence of supplemental natural feed sources, while the 50% FM replacement level provides sufficient nutrition. This combination of semi-natural conditions, protection from predators, and a balanced diet enables Nile tilapia to thrive, maximizing survival rates in these systems.

### 4.4. The Well-Being or Condition Factor (CF) of Nile Tilapia Fish Under Different Feeding Regimes

The CF serves as an important indicator of fish health, providing insight into the robustness of Nile tilapia by relating their weight to length and giving a clearer picture of their overall fitness. It can be influenced by a number of factors, such as the type of culture system, diet composition, and environmental conditions. Across the different studies of Nile tilapia cultured with varying levels of BSFL replacing FM, the CF offers a perspective on how these systems compare in maintaining fish health and growth efficiency.

In comparing hapas set in ponds, the extended culture period in Ouko et al. [125] produced a CF of 1.92 ± 0.01, showing that the fish had ample time to mature and develop a healthy body composition. The semi-controlled nature of hapas in ponds allows fish access to natural nutrients within the pond while also benefiting from a managed diet. The combination of a moderate 50% FM replacement and a long culture period creates an environment that encourages gradual and consistent growth, resulting in a higher CF. By comparison, shorter-term studies like Rana et al. [124], though observing rapid growth, would have less time for the fish to develop muscle mass and fat reserves. Although Rana et al. do not explicitly report the CF, the rapid growth over a short 28-day period likely did not allow the fish to develop the same degree of robustness as seen in longer studies, especially considering the early growth phase is typically characterized by fast weight gain rather than body mass accumulation.

In systems that use 100% FM replacement, such as Nairuti et al. [126], the CF would be influenced by how well the BSFL could meet the nutritional demands of the fish. While the exact CF is not reported, the integration of hapas nets in an earthen pond likely mitigated any potential deficiencies from the diet, as fish could forage on natural pond nutrients like algae and small organisms, enhancing their overall health. The natural supplementation, combined with a substantial 72-day culture period, likely contributed to a CF comparable to that of Ouko et al. [125], where the fish were given ample time to develop a balanced body weight relative to their size. The use of hapas also ensures that fish are protected from external stressors such as predation, reducing energy expenditure on survival, which in turn allows more resources to be directed toward growth and body condition development.

The cage systems in Mathai et al. [127] and Munguti et al. [19] offer an interesting contrast. In Mathai et al. [127], with 75% FM replacement, the CF was lower at 6.33 ± 0.32, which can be attributed to several factors, including the higher level of BSFL in the diet and the extended 182-day culture period. The high FM replacement level might not have provided the same nutrient density, particularly in EFAs and amino acids, which are crucial for the fish to develop optimal body condition. Furthermore, cages, while allowing for natural water flow, may not provide the same level of environmental enrichment as hapas in ponds. In contrast, Munguti et al. [19], using a 50% FM replacement in the same cage environment, reported a more favorable CF of 1.67 ± 0.01. The lower replacement level likely allowed for a more balanced nutrient profile, supporting better fat accumulation and overall growth. The semi-natural conditions in the earthen pond may have contributed to this, but the cage system still limits the fish's ability to forage for supplemental natural nutrients compared to hapas, which might explain why the CFs in cage systems tend to be lower than those in hapas.

The highly controlled environments of glass aquaria and tanks provide an interesting case when evaluating CFs. In Fayed et al. [128], the use of 25% FM replacement led to a CF of 1.93 ± 0.01, reflecting a well-balanced diet that allows for robust growth in a controlled setting. The low FM replacement ensures that the diet is nutritionally rich, supporting optimal weight gain relative to the fish's size. However, the controlled environment of glass aquaria may restrict natural behaviors like foraging, which could contribute to a slight limitation in the development of body mass when compared to semi-natural systems like hapas or earthen ponds. Similarly, Tippayadara et al. [18], with a 100% FM replacement in glass tanks, maintained excellent growth control, but the absence of natural food sources combined with a complete reliance on BSFL may limit the fish's ability to develop an optimal CF, even though the diet was sufficient to support high survival and growth rates.

In the case of plastic tanks, Limbu et al. [129], with a 75% FM replacement, reported a CF of 1.51 ± 0.05, which suggests some limitation in growth, possibly due to the reduced nutrient availability from a higher reliance on BSFL. The artificial nature of plastic tanks provides fewer opportunities for fish to forage naturally or receive environmental stimulation, which could also contribute to lower body condition. However, Muin et al. [130], using 50% FM replacement, likely produced a more favorable CF, as the diet provided a more balanced nutrient profile that better supported the development of muscle and fat, even within the constraints of a fully artificial system. The shorter 56-day culture period in this study, while limiting the time for significant body mass accumulation, likely allowed for a more focused period of rapid growth without the depletion of body reserves, which can sometimes occur in longer studies.

The comparison of CFs across these different aquaculture systems reveals that hapas in earthen ponds stand out as the most conducive to supporting a healthy and balanced CF. The semi-natural environment, combined with moderate FM replacement levels (as seen in Ouko et al. [125]), allows fish to benefit from both controlled feeding and natural food sources, resulting in a well-rounded development. While tank-based systems offer stable growth, the lack of environmental enrichment and reliance on higher levels of BSFL may limit the fish's ability to achieve the same robustness in body condition. Therefore, hapas in earthen ponds, particularly with a 50% FM replacement, provide the optimal conditions for maintaining a high CF, combining both environmental benefits and nutritional balance.

## 5. Sustainable Culture Systems for Scaling Up Nile Tilapia Production

### 5.1. Aquaponics

The use of BSFLM in aquaculture, particularly in aquaponics, has emerged as a promising strategy to enhance sustainability and resource efficiency. Aquaponics, a food production system that integrates aquaculture with hydroponics, offers a unique opportunity to reuse nutrient-rich water from fish farming to support plant growth [136]. The closed-loop nature of aquaponics minimizes waste while promoting efficient nutrient recycling, making it an attractive model for sustainable agriculture. The inclusion of BSFL as a feed source in this system further amplifies its sustainability potential. BSFL can be reared on organic waste, converting low-value byproducts into high-protein feed, which not only provides a cost-effective alternative to traditional FM but also addresses waste management issues [137].

Several studies have explored the integration of BSFLM in aquaponic systems, focusing on fish growth, nutrient recycling, and the overall performance of the system. Pinho et al. [137] conducted a comparative study in Europe to assess the impact of BSFL-based and FM-based diets on nutrient use and plant growth in decoupled aquaponic systems. Their findings showed that BSFL-based diets resulted in lower sodium concentrations in the nutrient solution compared to FM diets, which is beneficial for freshwater crops that are sensitive to high sodium levels. This highlights a key advantage of using BSFLM in aquaponics, as the accumulation of undesirable nutrients like sodium can hinder plant growth and overall system efficiency. Additionally, Pinho et al. [137] observed a 32% reduction in the need for inorganic fertilizers in aquaponic systems using BSFL-based feeds, further emphasizing the resource-saving potential of this approach. While the primary focus of the study was on plant performance, the implications for fish health and water quality are significant. The reduction in sodium levels and fertilizer input points to improved system sustainability, making BSFLM a valuable alternative to FM in aquaponics.

The potential of BSFLM to support sustainable aquaponics extends beyond their role as a feed source for fish. The byproduct of BSFLM production, known as frass, is a mineral-rich fertilizer that has shown promise in enhancing plant growth. A study done in North America by Romano and Islam [138], which tested BSF frass in aquaponic systems containing channel catfish, found that it provided an excellent source of nutrients for plants, closing the nutrient loop within the system. This boosts plant productivity and reduces the need for external fertilizers, aligning with the principles of circularity and sustainability that aquaponics seeks to achieve. Aquaponic systems can minimize waste, enhance nutrient recycling, and improve overall system efficiency by utilizing both BSFLM and their byproducts.

The use of BSFLM in aquaponic systems has also been investigated in relation to nutrient dynamics and fish growth in Europe. Gebauer et al. [139] explored the impact of different aquafeeds, including BSFLM, on nutrient efflux in aquaponic systems. Their study revealed that fish-fed BSFLM exhibited higher efflux of essential nutrients like magnesium and potassium, which are crucial for plant growth. This suggests that BSF-based diets not only meet the nutritional needs of fish but also enhance the nutrient profile of the water, benefiting both fish and plants in integrated systems. Similarly, Shaw et al. [7] evaluated the performance of Nile tilapia fed on BSFLM in an aquaponic system, comparing it to other protein sources such as poultry byproduct meal (PM) and FM. Although the study found that BSF meal was not as effective as FM in promoting rapid tilapia growth, it contributed valuable nutrients to the aquaponic water, particularly potassium, magnesium, and Cu. These nutrients play a vital role in plant health, reinforcing the importance of considering nutrient recycling when evaluating feed sources in aquaponics.

The potential of BSFL to support sustainable aquaponics lies in their ability to enhance nutrient cycling and reduce reliance on external inputs. The findings from these studies underscore the broader benefits of incorporating BSFL in aquaponic systems, where the focus is not only on fish growth but also on the efficient use of resources. As aquaculture systems continue to evolve, BSFLM offers a viable alternative to traditional FM, reducing the environmental impact of feed production while simultaneously improving system performance. Their ability to process waste and produce high-quality feed, along with the potential for their byproducts to enhance plant growth, makes them an integral part of a sustainable aquaponics framework.

### 5.2. RAS

RAS are increasingly gaining prominence in aquaculture due to their advanced waste management capabilities, particularly in the efficient removal of solid waste when compared to conventional flow-through systems. RAS is a closed-loop system where water is continuously filtered and reused, significantly reducing water consumption and minimizing environmental impacts, such as nutrient pollution and organic waste discharge. This system's ability to precisely control key water quality parameters, including temperature, oxygen levels, and waste accumulation, makes it highly effective in optimizing the growth, health, and overall productivity of fish.

The RAS is also aligned with the principles of the circular economy, which focuses on resource efficiency, waste reduction, and enhancing economic resilience within supply chains. One innovative approach in this context is the integration of RAS with insect-based feed production, particularly through the use of BSFLM. BSFLM can convert organic waste, such as fish feces and uneaten feed, into valuable insect biomass that can be repurposed as a protein source in fish diets. This reduces waste within the system and offers economic benefits by lowering the dependency on traditional protein sources like FM. However, when employing insect-based feeds in aquaculture, regulatory frameworks such as the European Union's Commission Regulation (EU) 2017/893 must be adhered to. These regulations govern the permissible substrates used for rearing insects and set safety limits for contaminants, such as heavy metals, ensuring that the insects destined for animal feed are safe and compliant.

In a European study by Yakti et al. [140], Nile tilapia were reared in RAS and fed four experimental diets that included FM, poultry blood meal (PBM), BSFLM, and PM as the primary protein sources. The study explored the potential of using fish waste as feed for BSFLM, demonstrating that the larvae's nutritional composition could be modulated by adjusting the composition of the fish diets. This finding underscores the interconnectedness between fish feed, fish growth performance, and the compositional quality of the BSFLM reared on the resulting waste. Such studies illustrate the dual functionality of RAS, where both fish production and waste valorization through insect farming are enhanced, creating a more sustainable and efficient system.

Another study by Khan et al. [141], conducted in Asia, evaluated the impact of BSFLM on the growth performance of Nile tilapia within a RAS. The study revealed that diets incorporating BSFLM supported robust fish growth, with lower levels of BSFL inclusion yielding better growth outcomes compared to higher inclusion levels. Additionally, as BSFLM levels increased, protein content in the fish declined while fat content increased, highlighting the need to carefully optimize BSFLM inclusion levels to maintain both growth performance and nutrient balance in RAS. This study further emphasized the potential of BSFLM as a sustainable alternative to FM, particularly in closed systems like RAS, where nutrient cycling and waste management are critical to long-term sustainability.

### 5.3. Fish Ponds

Fish ponds are the most widely used aquaculture systems for Nile tilapia worldwide, owing to their versatility, ease of management, and adaptability to different environments [6]. Ponds can be categorized into various types, including earthen, lined, and concrete ponds, each offering distinct advantages in terms of water retention, cost, and management practices. The multifunctional nature of pond systems, which allow for nutrient recycling, natural food availability, and integration with other agricultural practices, has been long recognized as a sustainable method for fish farming. In addition, fish ponds provide a semi-natural environment that can enhance fish growth by allowing access to both formulated feed and naturally occurring organisms such as plankton and algae [142].

Despite their prevalence in aquaculture, there is currently a lack of experimental studies that have specifically evaluated the performance of Nile tilapia fed with BSFLM in pond systems. This is likely due to the fact that BSFLM as a fish feed ingredient is still in its early stages of development, and most existing studies have focused on more controlled environments to accurately assess feed efficiency and growth performance. The relatively uncontrolled nature of pond systems, where environmental factors such as water quality and nutrient availability can fluctuate significantly, makes it more challenging to isolate the effects of alternative feeds like BSFLM in these systems.

### 5.4. Fish Cages

Fish cages are widely utilized aquaculture systems for Nile tilapia [143], particularly in regions where water bodies like lakes, rivers, and reservoirs provide an ideal environment for this type of culture. Cage aquaculture offers a versatile and scalable solution, allowing for the efficient use of natural water resources while supporting high-density fish farming. In Kenya, for instance, cage culture has become increasingly popular, especially around Lake Victoria, where small-to-large-scale fish farmers employ this method to meet the growing demand for tilapia [144]. The ease of installation and relatively low capital investment [145, 146], along with the efficient use of space in natural water bodies, make fish cages a practical choice for many aquaculturists globally [147].

Despite the fact that there are no studies documented evaluating the performance of Nile tilapia in cages, one of the key advantages of fish cages is their ability to facilitate water exchange, ensuring a constant flow of oxygen and nutrients while removing waste from the cages, which helps maintain good water quality. This natural water flow mimics the fish's wild habitat, potentially reducing stress and promoting better growth [148]. Moreover, cages can be easily monitored, making it convenient for farmers to manage stocking densities, feed applications, and fish health.

In the context of BSFLM as an FM alternative, fish cages offer several advantages that could enhance the performance of BSFL-based feeds. The continuous water flow in cages supports the efficient breakdown and dispersion of organic waste, which is critical when using high-protein feeds like BSFLM. The semi-natural environment provided by cages also allows tilapia to engage in natural feeding behaviors, potentially optimizing FCR when BSFLM is included in the diet. Additionally, the natural foraging that occurs in cages may complement BSFL-based diets, as the fish have access to both supplementary feed and naturally occurring organisms in the water, which could enhance their overall growth performance and health.

Given the growing interest in sustainable feed alternatives, incorporating BSFLM into cage-based Nile tilapia farming holds promise for reducing reliance on conventional FM while maintaining or even improving growth rates and feed efficiency. However, research is needed to further assess the specific impacts of BSFLM in cage aquaculture, particularly in terms of long-term fish health, water quality management, and economic viability.

## 6. Conclusion

This review highlights that while experimental aquaculture systems provide valuable insights into the use of BSFLM as an alternative feed for Nile tilapia, the performance of these feeds varies significantly across different culture systems. Hapas set in ponds consistently outperform other systems in terms of growth efficiency, feed utilization, and survival due to the combination of natural environmental enrichment and controlled feeding. These systems offer a balanced approach by providing access to both formulated feed and natural food sources, resulting in superior performance for short- and long-term culture. Cage systems in earthen ponds also perform well in survival rates but show lower growth and feed conversion efficiency. Conversely, tank-based systems like plastic tanks and glass aquaria provide precise control over environmental factors but lack the nutrient supplementation that enhances performance in pond-based systems. Based on experimental units, hapas in ponds most closely mimic large-scale earthen ponds, suggesting that earthen ponds may perform similarly well due to their natural food availability and nutrient cycling. Overall, hapas set in ponds stand out as the most effective and sustainable option.

However, expanding research and production in larger, commercial systems such as earthen ponds, RAS, aquaponics, and cages will be essential for optimizing the use of BSFLM in global aquaculture. Innovative processing technologies, including solvent extraction, mechanical pressing, and SFE, play a critical role in enhancing the nutritional quality and digestibility of BSFLM, thereby driving its potential use in aquafeeds. These technologies enable the efficient removal of excess lipids and the tailoring of fatty acid profiles to meet the specific dietary requirements of Nile tilapia, ensuring optimal growth performance and health.

Based on the findings, it is essential to tailor BSFLM feed formulations to meet the specific nutrient requirements of Nile tilapia across various production systems, necessitating detailed nutritional analyses and the application of innovative processing techniques to enhance feed quality and digestibility. Furthermore, regulatory compliance is paramount for the widespread adoption of BSFLM in aquaculture. For instance, the EU has implemented regulations to permit the use of insect-derived processed animal proteins (PAPs) in animal feed, specifically for nonruminant species like poultry, pigs, and aquaculture (Commission Regulation (EU) 2021/1372, 2021). Adhering to these regulations ensures that BSFLM can be integrated into commercial feeds without legal or safety concerns. Additionally, the scalability of BSFLM production must be addressed through the development of industrial strategies that optimize rearing conditions, improve feed conversion efficiency, and implement automation in production processes. The challenge of producing BSFLM in adequate volumes to warrant industrial processing remains significant. Overcoming this challenge requires investment in large-scale rearing facilities, advancements in automation technologies, and the establishment of efficient supply chains to meet the growing demand for sustainable aquafeed ingredients.

Collaborative partnerships involving academia, industry stakeholders, and governmental entities are crucial for advancing research and development, thereby overcoming challenges related to regulatory compliance and market integration. Additionally, further investigation is warranted to elucidate the role of BSFLM in diverse environmental contexts and to explore potential synergistic effects with other feed ingredients, which may enhance nutrient absorption and overall feed performance. Finally, aligning the adoption of BSFLM with global sustainability objectives is imperative, focusing on mitigating dependence on conventional feed sources and minimizing the environmental impacts of aquaculture practices. The integration of BSFLM into aquaculture systems holds significant promise for promoting sustainable intensification and enhancing food security while concurrently preserving marine ecosystems.

## Figures and Tables

**Table 1:** Primary macronutrient composition of BSFLM compared to the dietary requirements of Nile tilapia at various life stages.

Nutrient	Nutritional levels in BSFLM	Nile tilapia requirement
	**Source**: [20–22]	**Source:** [23, 24]
Protein	29.9%–48.2%	Fry: 30%–56% Juveniles: 30%–40% Adults: 28%–30%
Lipid	25.69%–28.43%	Fry: 4.4%–5.2% Adults: 6%–8%
Crude fiber	9.48%–9.96%	Optimal levels should be low for better digestion
Moisture	3.21%–7.10%	Not specified, but lower moisture content is preferable for storage
Ash	6.7%–15.4%	High ash can dilute energy density, but important for mineral supply

Abbreviation: BSFLM, black soldier fly larvae meal.

**Table 2:** Comparison of vitamin levels in BSFLM and Nile tilapia requirements.

Vitamin	Nutritional levels in BSFLM (mg/100g)	Nile tilapia requirement (mg/100g)
	**Source:** [20, 30, 31]	**Source:** [32–39]
Vitamin B1 (thiamine)	0.24–2.00	0.4
Vitamin B2 (riboflavin)	1.62–24.74	1
Vitamin C (ascorbic acid)	0.19–0.37	5–42
Vitamin E	0.62–1.30	5–10
Pantothenic acid (B5)	3.85	1
Niacin (vitamin B3)	7.10	2.6–12.1
Pyridoxine (vitamin B6)	0.60	0.17–1.65
Folic acid	0.27	0.05
Biotin	0.035	0.006
Vitamin B12	0.00558	Not required
Choline	110	100

Abbreviation: BSFLM, black soldier fly larvae meal.

**Table 3:** Mineral content of BSFLM compared to the dietary requirements of Nile tilapia.

Mineral	Nutritional levels in BSFLM (mg/kg)	Nile tilapia requirement (mg/kg)
	**Source:** [20]	**Source:** [23, 32, 58, 66, 69, 75, 76, 79, 83, 84]
Calcium	13,005.12–26,515.88	3500–4200
Phosphorus	6000–9100	9000
Potassium	11,256.11–13,156.34	2000–3000
Sodium	3385.20–5028.42	Not specified
Magnesium	2505.98–3616.55	590
Zinc	303.07–1209.63	30–80
Iron	300.75–689.24	30–170
Manganese	134.93–162.51	13–15
Copper	13.69–29.10	3–5

Abbreviation: BSFLM, black soldier fly larvae meal.

**Table 4:** Essential amino acid composition ranges for BSFLM (whether fatted or defatted) and optimum amino acid requirement for Nile tilapia.

Amino Acid	BSFLM Range (% DM)	Optimum Nile tilapia requirement
Arginine	1.80–5.64	1.36–4.20
Histidine	1.18–4.63	0.47–1.72
Isoleucine	1.59–2.40	0.88–3.11
Leucine	2.52–8.80	1.33–3.39
Lysine	1.91–7.03	1.56–5.12
Methionine	0.53–2.74	0.65–2.68
Phenylalanine	1.35–6.24	1.01–3.75
Threonine	1.42–4.48	1.45–3.75
Valine	2.29–5.80	0.96–2.80

Abbreviation: BSFLM, black soldier fly larvae meal.

**Table 5:** Growth performance parameters in various aquaculture systems.

Culture systems	Fishmeal replacement level with BSFLM (%)	Culture period (days)	Initial fish weight	Final fish weight	Weight gained (g)	Specific growth rate	Feed conversion ratio (FCR)	Survival rate (%)	Condition factor	Reference
Hapas set in ponds	50	28	0.99 ± 0.17	30.71 ± 1.06	29.72 ± 0.95	3.743 ± 0.35	1.7 ± 0.2	93.33	*⁣* ^*∗∗*^	Rana et al. [124]
Hapas set in ponds	50	147	0.11 ± 0.01	23.20 ± 1.05	23.09 ± 1.15	1.31 ± 0.03	1.57 ± 0.02	96.74 ± 3.18	1.92 ± 0.01	Ouko et al. [125]
Hapas set in ponds	100	72	52.2 ± 0.74	112.8 ± 2.62	60.5 ± 2.11	1.1 ± 0.02	1.1 ± 0.01	98.3 ± 1.67	*⁣* ^*∗∗*^	Nairuti et al. [126]
Cages built in an earthen pond	75	182	33.88 ± 2.18	98.86 ± 3.35	64.98 ± 2.88	0.52 ± 0.02	2.13 ± 0.06	95.83 ± 3.31	6.33 ± 0.32	Mathai et al. [127]
Cages built in an earthen pond	50	182	24.74 ± 0.75	76.90 ± 4.80	52.16 ± 5.02	*⁣* ^*∗∗*^	*⁣* ^*∗∗*^	100	*⁣* ^*∗∗*^	[86]
Cages built in an earthen pond	50	182	24.72 ± 0.39	76.95 ± 2.52	52.22 ± 2.58	0.60 ± 0.02	1.46 ± 0.16	100	1.67 ± 0.01	[19]
Glass tanks	25	84	4.25 ± 0.08	20.60 ± 0.31	16.30 ± 0.39	0.82 ± 0.001	1.22 ± 0.005	98.00 ± 0.58	1.93 ± 0.01	[128]
Glass tanks	100	84	14.51 ± 2.06	43.02 ± 3.15	28.50 ± 3.37	1.30 ± 0.17	2.23 ± 0.15	100	*⁣* ^*∗∗*^	Tippayadara et al. [18]
Plastic tanks	75	84	0.001	3.661 ± 0.27	3.66 ± 0.27	2.15 ± 0.03	0.89 ± 0.02	96.46 ± 1.61	1.51 ± 0.05	Limbu et al. [129]
Plastic tanks	50	56	3.03 ± 0.05	11.77 ± 0.16	8.74 ± 0.18	2.43 ± 0.04	2.91 ± 0.10	100	*⁣* ^*∗∗*^	Muin et al. [130]

*Note*: The *⁣*^*∗∗*^ symbol indicates that the study did not evaluate those parameters.

Abbreviations: BSFLM, black soldier fly larvae meal; FCR, feed conversion ratio.

## Data Availability

Data sharing does not apply to this article as no new data are created or analyzed in this study.
